# HPLC with Fluorescence and Photodiode Array Detection for Quantifying Capmatinib in Biological Samples: Application to In Vivo and In Vitro Studies

**DOI:** 10.3390/molecules27238582

**Published:** 2022-12-05

**Authors:** Aref Zayed, Sana’a A. Jaber, Jomana Al Hroot, Sahar Hawamdeh, Nehad M. Ayoub, Nidal A. Qinna

**Affiliations:** 1 Department of Medicinal Chemistry and Pharmacognosy, Faculty of Pharmacy, Jordan University of Science and Technology (JUST), Irbid 22110, Jordan; 2 Department of Clinical Pharmacy, Faculty of Pharmacy, Jordan University of Science and Technology (JUST), Irbid 22110, Jordan; 3 University of Petra Pharmaceutical Center (UPPC), Faculty of Pharmacy and Medical Sciences, University of Petra, Amman 11196, Jordan

**Keywords:** capmatinib, HPLC, pharmacokinetics, fluorescence, rat plasma, microsomes, method development, metabolic stability, tyrosine kinase inhibitor

## Abstract

Capmatinib, a recently approved tyrosine kinase inhibitor, is used for the treatment of non-small cell lung cancer. We describe two new HPLC methods for capmatinib quantification in vivo and in vitro. HPLC with a fluorescence detection method was used to quantify capmatinib in plasma for the first time. The method was successfully applied in a pharmacokinetic study following a 10 mg/kg oral dose of capmatinib given to rats. The chromatographic separation was performed using a Eurospher II 100-3 C18H (50 × 4 mm, 3 µm) column and a mobile phase containing 10 mM of ammonium acetate buffer (pH 5.5): acetonitrile (70:30, *v*/*v*), at a flow rate of 2.0 mL min^−1^. The study also describes the use of HPLC-PDA for the first time for the determination of capmatinib in human liver microsomes and describes its application to study its metabolic stability in vitro. Our results were in agreement with those reported using LC-MS/MS, demonstrating the reliability of the method. The study utilized a Gemini-NX C18 column and a mobile phase containing methanol: 20 mM ammonium formate buffer pH 3.5 (53:47, *v*/*v*), delivered at a flow rate of 1.1 mL min^−1^. These methods are suitable for supporting pharmacokinetic studies, particularly in bioanalytical labs lacking LC-MS/MS capabilities.

## 1. Introduction

Lung cancer is a leading cause of death among cancer patients and the second most diagnosed cancer around the world [1]. It has two major histopathologic subtypes: small-cell lung cancer (SCLC) and non-small cell lung cancer (NSCLC). The latter accounts for 85% of all lung cancer cases.

Lung cancer treatment depends on many factors, including disease stage, tumor histology, and gene mutations. Determining the presence of such mutations is critically important to tailor effective individualized treatment [2]. NSCLC patients have had gene mutations that might be targeted.

Mesenchymal Epithelial Transition factor receptor (MET) is involved in a variety of important biological functions that involve cell development, proliferation, and migration. Mutations in MET, however, are associated with tumor cell proliferation and metastasis, and therefore, MET was a potential target for the development of novel cancer therapy drugs. Several MET tyrosine kinase inhibitors (TKIs) were approved for the treatment of NSCLC patients with specific MET mutations [3].

**Figure 1:**
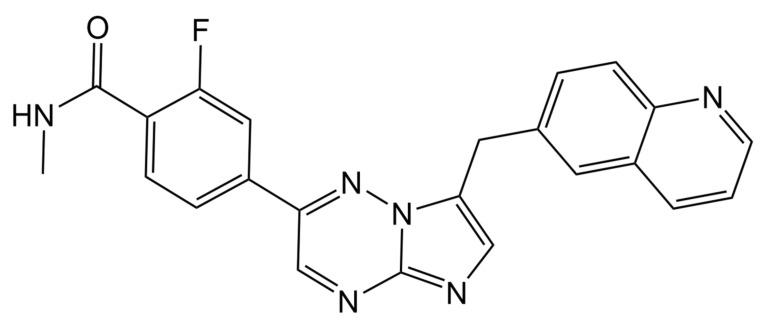
Capmatinib chemical structure.

Capmatinib (Figure 1), a TKI, was approved by the U.S. Food and Drug Administration (FDA) in 2020 to treat metastatic NSCLC patients with MET exon 14 skipping mutation [4]. Capmatinib was found to be highly selective for MET in vitro [5], and it was clinically found to be a compelling treatment and was well tolerated in patients with MET mutation [6,7].

Capmatinib is orally bioavailable with a maximum concentration of approximately 3800 ng/mL after 2 h of administering a 600 mg dose [8]. It is mainly metabolized in the liver by cytochrome P450 (CYP) 3A4 and aldehyde oxidase, with subsequent renal and biliary excretion [8,9]

The quantification of capmatinib in biological samples in vivo and in vitro is necessary to investigate its pharmacokinetics and potential interactions with other drugs, diet, and supplements. The chromatographic methods for the determination of the capmatinib concentration in biological samples were predominantly performed by LC-MS/MS [8,10] and UPLC-MS/MS [11]. Zhou et al., developed and validated a fast and reliable UPLC-MS/MS for the determination of capmatinib plasma levels in rats following three oral doses. The method, which has a working range of 1–4000 ng/mL, was applied successfully to investigate the pharmacokinetics of capmatinib in vivo [11]. Another study reported the use of LC-MS/MS for the quantification of capmatinib in rat plasma in preclinical studies. The method had a working range of 1–2000 ng/mL [10]. A recently published study by Ali et al. investigated the photophysical and fluorescence characteristics of capmatinib under various experimental conditions of solvents, pH, and temperature, using spectrofluorimetry, with no chromatography [12]. The study also reported HPLC with the photodiode array detection (HPLC-PDA) method for capmatinib quantification using a spiked serum matrix.

In this study, we report the development and validation of the first HPLC with the fluorescence detection (HPLC-FLD) method for the quantification of capmatinib in biological samples. In addition, the application of the developed method in a pharmacokinetic study to measure in vivo plasma levels of capmatinib is reported. The developed HPLC-FLD method offers excellent selectivity and sensitivity and has the advantages of simplicity and lower capital cost compared to LC-MS/MS. Our study is also the first to report a simple HPLC with a photodiode array detector (PDA) method for quantifying capmatinib in human liver microsomes (HLMs). This method was used to estimate capmatinib in vitro half-life and intrinsic clearance, demonstrating its sensitivity and reliability for in vitro metabolic assays. Collectively, the methods reported in this study are appealing for those supporting the preclinical and pharmacokinetic (PK) studies involving capmatinib, such as bioavailability, metabolic, and drug interaction studies.

## 2. Results and Discussion

### 2.1. Method Development

The overall objective of the method development experiments was to obtain simple, rapid, and sensitive methods that are suitable for quantifying capmatinib in rat plasma and liver microsomes. Preclinical and pharmacokinetic studies often require the quantification of trace levels of the drug in a high number of samples to obtain reliable data. We, therefore, aimed to select simple extraction procedures and to develop short-run-time chromatographic separations for the analytical methods.

LC-MS/MS is the gold standard in bioanalysis for preclinical and pharmacokinetic studies. HPLC-FLD, however, offers a simpler and more economical alternative while having an excellent sensitivity and selectivity. Such a technique is vital for labs with a limited budget and lacking LC-MS/MS capabilities. Our HPLC-FLD method was validated with a lower limit of quantification (LLOQ) of 19.5 ng/mL as it was sufficient for the in vivo study. However, the method has the potential to produce a significantly lower LLOQ by skipping the dilution step we deliberately included (200 μL diluted to 600 μL) to improve the column working life by reducing the amount of injected matrix. Furthermore, the addition of a preconcentration/evaporation step, if needed, fits well with the extraction method used and would further improve the sensitivity to match that of the two reported LC-MS/MS methods which have an LLOQ = 1 ng/mL in rat plasma [10,11]. This sensitivity, however, was not required for the in vivo and in vitro samples tested in the present study and therefore was not investigated.

Due to the expected low concentration of capmatinib in plasma in the in vivo study, and since capmatinib possesses a quinoline moiety that is known to be fluorescent [13,14], we decided to use fluorescence detection to exploit its excellent sensitivity and high selectivity, which are relevant when quantifying trace levels of drugs in complex biological matrices. The selectivity of the method was further enhanced due to the chromatographic separation adding another analytical dimension by which to resolve potential matrix interferences.

For the chromatographic separation, we examined several stationary phases and mobile phase conditions to obtain an efficient separation. The best separation was achieved using an endcapped C_18_ stationary phase (Eurospher II 100-3 C18 H) with a mobile phase consisting of 30:70 *v*/*v* acetonitrile and 10 mM of ammonium acetate buffer (pH 5.5). The endcapping and the efficient 3 µm particles of the stationary phase resulted in sharp peak shapes and excellent resolution, which allowed for the increasing of the flow rate to 2 mL/min and thus the doubling of the speed of the analysis without sacrificing the separation. The chromatographic parameters of the optimized HPLC-FLD method are presented in Table 1. 

**Figure 2:**
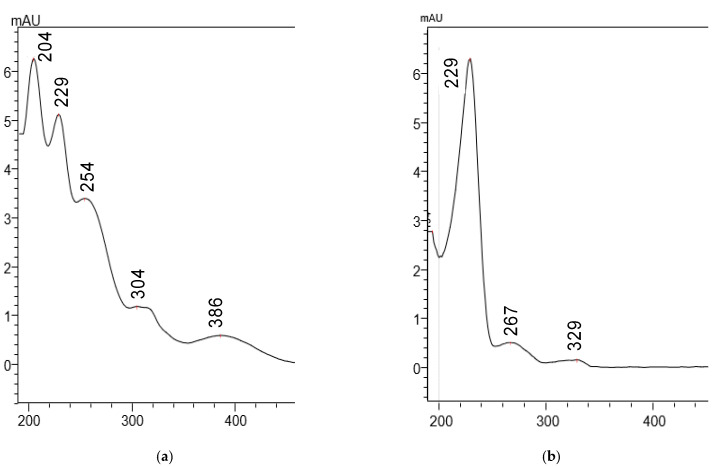
(**a**) Capmatinib absorption spectrum; (**b**) naproxen (internal standard) absorption spectrum. Spectra were obtained by the photodiode array detection at the corresponding retention times of capmatinib (3.7 min) and naproxen (8.9 min) following injection of individual standard solutions (1.3 and 1.0 mg/mL, respectively) on the HPLC-PDA system.

**Figure 3:**
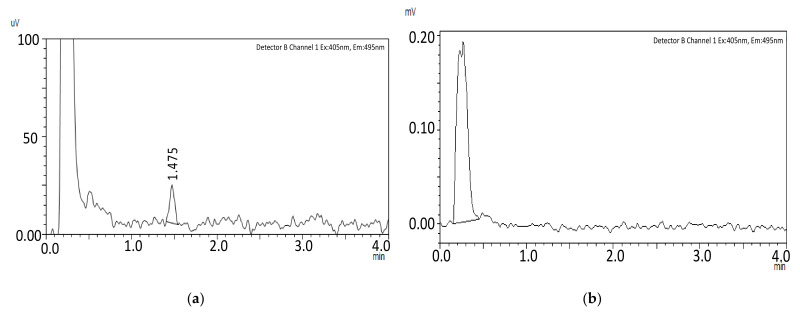

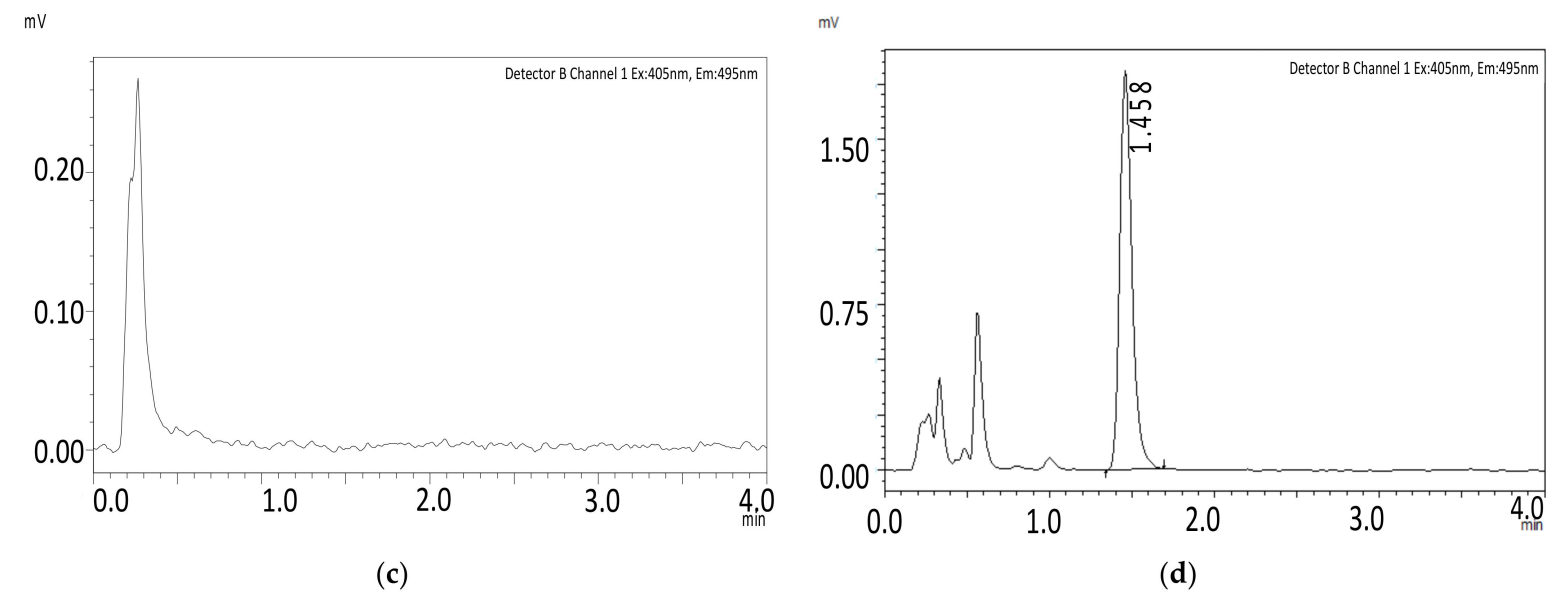
Representative HPLC-FLD chromatograms at excitation and emission wavelengths at 405 and 495 nm, respectively, for (**a**) plasma sample spiked with capmatinib (t_R_ = 1.47 min) at LLOQ level (19.5 ng/mL); (**b**) blank plasma sample; (**c**) in vivo pre-dose plasma sample; (**d**) in vivo plasma sample obtained 1 h after oral administration of capmatinib to rat, concentration = 1700 ng/mL.

Different excitation and emission wavelengths relevant to quinoline-containing compounds were experimented with [13,14]. The highest signal intensity was achieved using excitation and emission wavelengths at 405 and 495 nm, respectively. The absorption plot of capmatinib (Figure 2) shows a λ_max_ at 390 nm, which is close to the excitation wavelength selected for the method at 405 nm. The selected emission wavelength at 495 nm is close to that reported for quinoline at 500 nm. For the internal standard warfarin, we used the reported excitation and emission wavelengths at 310 and 390 nm, respectively [15]. The representative chromatograms obtained using the final method are shown in Figure 3. 

For the microsomal incubation experiments, an HPLC method with PDA detection was developed since the capmatinib levels in microsomal in vitro samples are relatively higher than those in plasma. Moreover, HPLC-PDA is a very common analytical tool and widely available in research laboratories. To the best of our knowledge, no HPLC-PDA methods were previously reported for quantifying capmatinib in the liver microsomes matrix.

To aid in the method development, and due to the expected interferences detectable by PDA, a wide pH range Gemini NX C_18_ column was used, to experiment with various pH conditions and buffers to offer flexibility in manipulating and resolving the chromatographic peaks. Methanol: ammonium formate (20 mM, pH 3.5) (53:47, *v*/*v*) was selected as a mobile phase. When choosing an internal standard, and since the PDA detector is not as selective as the fluorescent or mass spectrometry detectors, only compounds that eluted after capmatinib were considered. The internal standard could otherwise coelute with the early eluting polar metabolites or matrix interferences. Naproxen was a good choice, particularly in the acidic conditions which suppressed its ionization and thus provided good retention. The naproxen absorption spectra are shown in Figure 2b. The chromatographic parameters of the optimized HPLC-PDA method are presented in Table 2.

### 2.2. Method Validation

#### 2.2.1. HPLC-FLD Method Validation

Following the successful method development and optimization experiments, a validation for the HPLC-FLD method was conducted according to the European Medicines Agency (EMA) guidelines [16]. The validation parameters were linearity, LLOQ, accuracy, precision, selectivity, and carryover. Capmatinib stability in rat plasma was thoroughly investigated in previous studies [10,11].

##### Linearity and LLOQ

The capmatinib calibration curve demonstrated excellent linearity (mean *R^2^* > 0.99) in the concentration range of 19.5–3920 ng/mL. The linearity parameters are presented in Table 3. The LLOQ = 19.5 ng/mL was set to reflect the expected lowest concentration after the oral administration although the method is capable of quantifying significantly lower concentrations. Figure 3a shows a chromatogram for a plasma sample spiked with capmatinib at the LLOQ level (19.5 ng/mL(. The high sensitivity of the method allowed for a dilution step for the sample supernatants (200 µL) with a relatively large sample solvent (400 µL) volume before injection, to reduce the amount of matrix injected on the column and to improve its longevity.

##### Accuracy and Precision

The intra-day RSD values were in the range of 4.15–9.57%, and the values of e_r_ were between 5.15 and 8.06%. The inter-day RSD values were in the range of 7.2–11.8%, and inter-day e_r_ values were between 5.55 and 10.95%. The results are presented in Table 4.

##### Selectivity

No interferences were detected in the retention time of capmatinib or warfarin when blank pooled plasma was analyzed (Figure 3b). Moreover, no capmatinib peak was observed in the pre-dose plasma samples obtained in the in vivo study (Figure 3c). 

##### Carryover

No peaks were detected when a blank sample was injected after a high-concentration sample, indicating that the method has no potential for carryover.

#### 2.2.2. HPLC-PDA Method Validation

**Figure 4:**
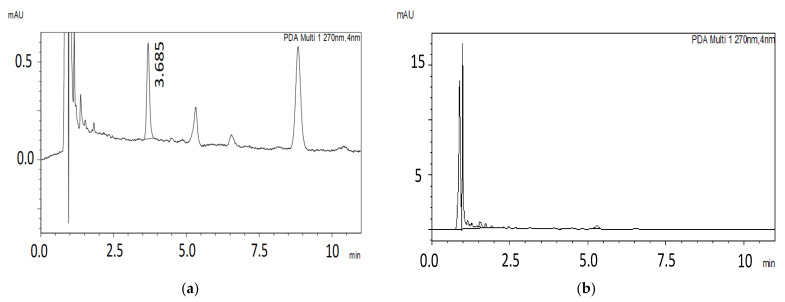
Representative chromatograms of (**a**) HLM sample spiked with capmatinib (t_R_ = 3.68 min) at LLOQ level 206 ng/mL at wavelength of 270 nm and (**b**) blank HLM sample at wavelengths of 270 nm.

The proposed HPLC-PDA was validated according to the EMA guidelines in terms of linearity, LLOQ, accuracy, precision, selectivity, and carryover. Figure 4 shows representative chromatograms obtained using the final HPLC-PDA method for human liver microsomes samples.

##### Linearity and LLOQ

The calibration curve for capmatinib showed excellent linearity (mean *R^2^* > 0.99) in the concentration range from 206 to 4124 ng/mL. Figure 4a shows a sample of microsomes spiked with capmatinib at the LLOQ level (206 ng/mL). The linearity parameters are presented in Table 5. LLOQ was set to reflect the expected lowest concentration at the end of the 1 h incubation time

##### Accuracy and Precision

The intra-day RSD values were in the range of 0.86–3.8%, and the e_r_ values were in the range of 2.4–14%. The inter-day RSD values were in the range of 7.7–9.1%, and the inter-day e_r_ values were in the range of 2.7–6.4%. The accuracy and precision results are summarized in Table 6.

##### Selectivity

No interferences were detected in the retention time of capmatinib or the internal standard naproxen when the blank HLM samples were analyzed (Figure 4b).

##### Carryover

No analyte or internal standard peaks were observed in their corresponding retention times when the blank samples were injected after the highest standard concentration, thus demonstrating that there was no carryover effect.

### 2.3. In Vivo Pharmacokinetic Study

**Figure 5:**
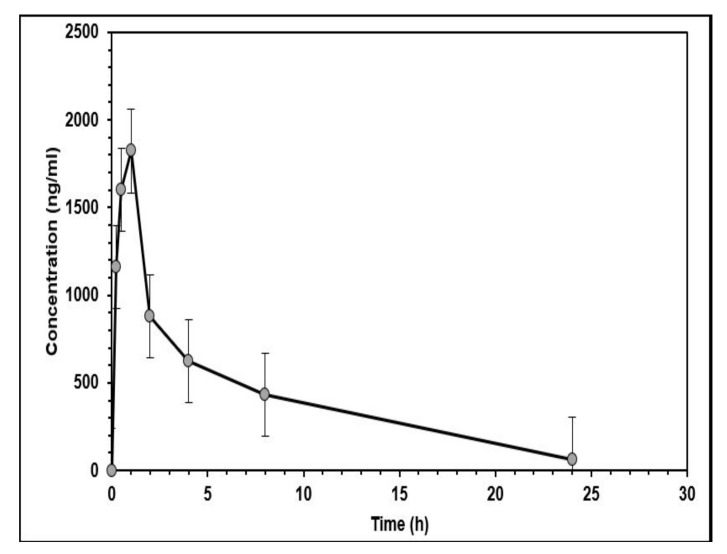
Plasma concentration–time profile of capmatinib after a single oral dose of 10 mg/kg in male SD rats (*n* = 6). The data are expressed as mean ±SD.

To demonstrate the reliability of the HPLC-FLD method in real settings, the validated method was used to determine capmatinib pharmacokinetics in male Sprague Dawley (SD) rats after the administration of a 10 mg/kg single oral dose of the drug. The method was successful in quantifying the capmatinib in vivo levels, and the PK profile was determined (Figure 5).

The extent of the capmatinib bioavailability, represented by ${\mathrm{AUC}}_{0}^{\inf}$, the time to reach the peak plasma concentration (T_max_), the half-life (t_1/2_), the apparent total clearance (CL/F), the apparent volume of distribution of the terminal phase (Vd/F), the total area under the concentration–time curve (${\mathrm{AUC}}_{0}^{\inf}$), and the mean residence time (${\mathrm{MRT}}_{0}^{\inf})$ were determined. The average peak plasma concentration was 1636.5 ± 509.6 ng/mL, which was reached after about 0.67 h. The pharmacokinetic parameters determined in the study are shown in Table 7. Samples were taken up to 24 h, where more than 99% of capmatinib had been eliminated (i.e., more than 10 half-lives). The capmatinib showed rapid absorption (T_max_ = 0.67 h), which was in agreement with similar studies in rats [11]. In comparison, studies in humans showed slower absorption, which could be due to species differences and/or the administration of capmatinib without excipients in the rats [11,17]. It was noticed that the peak plasma concentration in our study was lower than that reported in previous rat studies (~3800 ng/mL) [10,11], conducted by LC-MS/MS, possibly due to the use of different drug vehicles in drug administration. DMSO was used initially to dissolve capmatinib in these reported studies, whereas no DMSO and only 0.5% CMC were used in our study. Capmatinib has low solubility in aqueous media [18], which may have resulted in slower drug absorption and thus the lower bioavailability in this study [19].

### 2.4. In Vitro Metabolic Study

**Figure 6:**
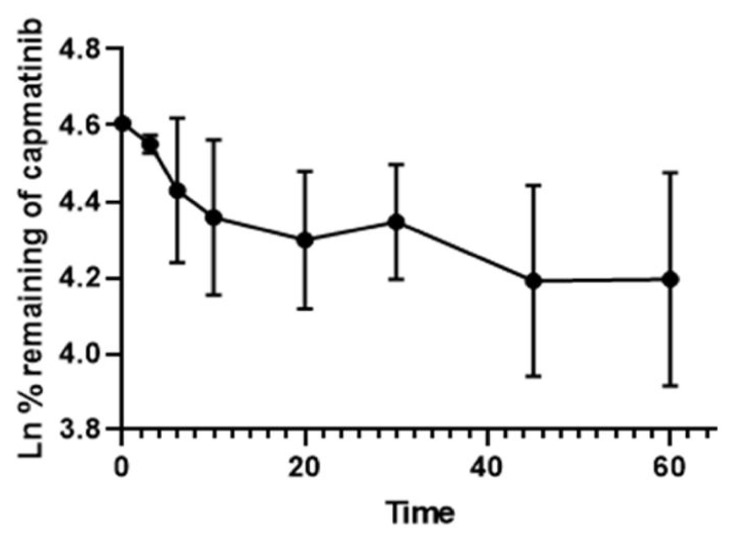
Capmatinib depletion rate when incubated with 0.3 mg/mL HLMs. The data are shown in mean ±SD.

The HPLC-PDA method developed in this work was applied successfully to evaluate the metabolic stability of capmatinib in vitro following incubation with human liver microsomes. We plotted the natural logarithm of the remaining capmatinib at each of the eight timepoints (0–60 min) against their corresponding incubation time. The results showed that the capmatinib depletion rate was linear (*R^2^* = 0.97) over the first 10 min (Figure 6) before it slowed down and reached a plateau at the end of the incubation. The *slope* of the linear part (−0.033), which represents the disappearance rate constant (*k*) of the capmatinib, was used to calculate the *in vitro t*_1/2_ according to the following equation:

$$t_{1/2}\;=\;\frac{Ln\;2}{Slope}$$

The intrinsic clearance of the capmatinib was calculated using the following equation:

(1) $$\mathrm{Intrinsic}\;\mathrm{Clearance}\;({CL}_{int})=\frac{0.693}{\;in\;vitro\;t_{1/2}}\times \frac{\mu l\;incubation\;}{mg\;microsomes}$$

The *in vitro t*_1/2_ and *CL_int_* were 21.1 min and 110.3 µL/min/mg, respectively. Our data are in agreement with those previously reported by LC-MS [18], thus demonstrating the reliability of the HPLC-PDA method and its fitness to evaluate the in vitro metabolic studies of capmatinib. The results showed that capmatinib is a high-clearance compound (>100 µL/mg/min) according to the classification typically used to describe a compound’s clearance based on its *in vitro t_1/2_* and *CL_int_* values [19,20]. The in vitro data obtained by our method are useful to predict in vivo human hepatic clearance and for the optimization of the drug’s oral bioavailability [21,22,23], particularly if combined with other agents that may affect capmatinib metabolism, which may produce potential drug interactions. 

## 3. Materials and Methods

### 3.1. Chemicals and Reagents

Capmatinib (purity 98.0%) was purchased from Cayman (Ann Arbor, MI, USA); warfarin (internal standard for the analysis of the in vivo samples) was purchased from Sigma-Aldrich (St. Louis, MO, USA); and naproxen (internal standard for the in vitro study) was kindly supplied as a gift from Hikma Pharmaceuticals (Amman, Jordan). HPLC grade acetonitrile, methanol, ammonium acetate, ammonium formate, and formic acid were purchased from Fisher Scientific (Waltham, MA, USA). HPLC-grade water was purchased from Honeywell (Charlotte, NC, USA). Nuclease-free water was obtained from Integrated DNA Technologies (Coralville, IA, USA). NADPH regenerating system solution A, NADPH regenerating system solution B, HLMs, and 0.5 M phosphate buffer were obtained from Corning (Ann Arbor, MI, USA).

### 3.2. Equipment

The separation and quantification were carried out using the Shimadzu LC-2040C 3D plus (Tokyo, Japan) HPLC system, equipped with either a fluorescence detector (FLD), RF-20A, or a photodiode array detector (PDA). The FLD was set at the excitation and emission wavelengths of 405 nm and 495 nm for the capmatinib, respectively, and 310 and 390 nm for the warfarin, respectively. The PDA detector monitored the capmatinib and naproxen at 270 and 232 nm, respectively. Data acquisition and integration were attained using LabSolutions software, Shimadzu Corporation, Kyoto, Japan (version 6.87 SP1).

### 3.3. Chromatographic Conditions

The HPLC-FLD method used for quantifying the in vivo levels of capmatinib in rat plasma employed a Eurospher II 100-3 C18 H (50 × 4 mm, 3 µm) column; the mobile phase was a mixture of acetonitrile and ammonium acetate buffer (10 mM, pH 5.5) (30:70, *v*/*v*) delivered at a flow rate of 2 mL/min. The column temperature was 25 °C, and the injection volume was 5 µL. For the quantification of capmatinib in human liver microsomes by HPLC-PDA, the method utilized a Gemini NX C18 column (100 × 4.6 mm, 3 µm) and a mobile phase consisting of methanol and ammonium formate buffer (20 mM, pH 3.5) (53:47, *v*/*v*) at a flow rate of 1.1 mL/min. The column temperature was 30 °C and the injection volume was 20 µL.

### 3.4. Preparation of Standard Solutions, Calibration, and Quality Control Samples

For the quantification of capmatinib in plasma by HPLC-FLD (in vivo study), a stock solution of capmatinib prepared by dissolving capmatinib in methanol to produce a concentration of 2 mg/mL (5 mM) was used. Nine working solutions were prepared from the stock solution (1.3, 6.5, 19.6, 32.6, 65.3, 98, 130.6, 196, and 261.3 µg/mL). The internal standard solution of warfarin had a concentration of 15 µg/mL in methanol. To prepare the calibration standards, we spiked 985 µL of blank pooled rat plasma with a volume of 15 µL of corresponding working solution to generate 9 non-zero calibration points: 19.5, 97.5, 294, 490, 979.5, 1470, 1960, 2940, and 3920 ng/mL, in addition to the blank samples. Quality control (QC) samples were prepared at 4 concentration levels, as given in the following: 19.5 ng/mL lower limit of quantification (LLOQ), 58.5 ng/mL QC-Low (QCL), 1470 ng/mL QC-Medium (QCM), and 3137 ng/mL QC-High (QCH).

For the quantification of capmatinib in human liver microsomes by HPLC-PDA (in vitro study), we prepared two capmatinib working solutions: working solution I, containing 1.44 mg/mL of capmatinib in methanol, and working solution II, containing 14.4 µg/mL of capmatinib in 100 mM phosphate buffer. To use as an internal standard, we prepared a stock solution of naproxen, 1 mg/mL, in methanol. We used two naproxen working solutions: working solution I, containing 100 µg/mL of naproxen in methanol, and working solution II, containing 1 µg/mL of naproxen in methanol. We added aliquots of the capmatinib working solution II to the human liver microsomes matrix and completed the volume to 1 mL by 100 mM phosphate buffer to generate eight non-zero calibration standards (206, 1237, 2474, 2886, 3000, 3500, 3900, and 4124 ng/mL). The quality control samples prepared separately at four concentration levels were: 206 ng/mL LLOQ, 618.6 ng/mL QCL, 2000 ng/mL QCM, and 3093 ng/mL QCH.

### 3.5. Sample Preparation

For plasma preparation, we added a volume of 10 µL of internal standard (15 µg/mL) to 100 µL of each calibration standard, QC, and the unknown samples before being mixed by a vortex. A volume of 300 µL of ice-cold acetonitrile was mixed with the samples for 30 s to precipitate proteins. The samples were then centrifuged for 10 min at 6000 rpm before transferring the supernatant and filtering it using a 0.22 µm syringe filter into clean test tubes. The samples were then diluted by taking 200 μL aliquots of the filtered solution and mixing each with 400 µL of ammonium acetate buffer (pH 5.5) before injecting 5 µL of the final sample solution into the HPLC-FLD system for quantification.

For the microsome sample preparation, we added a volume of 100 µL of ice-cold methanol containing the internal standard (working solution II) to 100 µL of the microsomal mixture following incubation and mixed it by vortex for 15 s before centrifugation at 10,000 rpm (5 min at 4 °C). The supernatant (100 µL) was then transferred to clean test tubes before adding and mixing it with 90 µL of ammonium formate buffer (pH 3.5). The sample solutions were then injected (20µL) into the HPLC-PDA system for quantification.

### 3.6. Method Validation

The HPLC methods were validated according to the European Medicines Agency (EMA) bioanalytical method validation guidelines in terms of linearity, the lower limit of quantification, accuracy, precision, selectivity, and carryover [16].

#### 3.6.1. Linearity and LLOQ

To assess linearity, we used at least three calibration curves to plot the peak area ratios of the capmatinib/internal standard against the concentrations of calibration standards. A weighting factor of 1/*x*^2^ was used in all the calibrations. The acceptance criterion for linearity was a value of 0.99 or greater for the coefficient of determination (*R^2^*).

#### 3.6.2. Accuracy and Precision

We used at least five replicates of QC samples in a single batch to assess intra-day accuracy and precision. A minimum of three runs was analyzed on at least two different days to evaluate the inter-day accuracy and precision. We calculated relative error (e_r_) at each QC concentration to estimate the accuracy and used relative standard deviation (RSD) to estimate the precision. Values within ± 15%, and not exceeding 20% for LLOQ, were considered as evidence of acceptable accuracy and precision.

#### 3.6.3. Selectivity

To assess the method selectivity, blank pooled plasma from 10 different rats was analyzed to check for potential interferences in the capmatinib retention time. Blank samples containing human liver microsomes only and phosphate buffer, with no drug or internal standard, were also prepared to confirm the lack of interferences from the sample in the retention times of capmatinib and the internal standard.

#### 3.6.4. Carryover

To assess carryover, the blank samples were run directly after the highest calibration standards. Triplicate runs were used, and the run was accepted if the carryover was not greater than 20% of LLOQ and 5% for the internal standard.

### 3.7. In Vivo Pharmacokinetic Study

#### 3.7.1. Animal Handling

Adult male Sprague Dawley (SD) rats were used with an average weight of 230 ± 23 g. The animals were accommodated at the animal facility of the University of Petra Pharmaceutical Center (Amman, Jordan) under a controlled temperature (22–24 °C), humidity (55–65%), and a 12 h light/dark photoperiod cycle. The rats were offered clean tap water ad libitum and fed with a standard pellet diet (Jordan Feed Co. Ltd., Amman, Jordan). The rats were randomized into groups and acclimatized for 10 days before experimentation.

#### 3.7.2. In Vivo Experimental Design

The rats were randomized, weighed, and tail marked into two groups, namely a capmatinib-administered group and a placebo group (*n =* 6). The capmatinib was freshly dissolved in a 0.5% carboxymethylcellulose (CMC) solution prepared in distilled water while the placebo solution consisted of 0.5% CMC alone. The fasting rats (12 h, water ad libitum) were administered either capmatinib at 10 mg/kg dose or an equivalent amount of 0.5% CMC solution by oral gavage using a stainless-steel oral gavage needle (Harvard Apparatus, Holliston, USA).

A volume of 200 µL of the blood samples was obtained from the retro-orbital plexus using clean capillary tubes and collected into Minicollect EDTA tubes. Blood sampling was performed under light isoflurane anesthesia (Hikma Pharmaceuticals, Amman, Jordan) (5% induction and 2.5% maintenance) carried by oxygen (dual-flow oxygen concentrator, China) using a low-flow anesthesia system (SomnoSuite, Kent Scientific, Torrington, CT, USA). An initial blood sample, a zero-time sample, was collected from each animal. The blood samples were then collected at specific time intervals at 0.25, 0.50, 1, 2, 4, 8, and 24 h post-drug administration. The collected blood tubes were centrifuged for 10 min at 6000 rpm using a Hettich EBA 20 centrifuge, Tuttlingen, Germany. The clear plasma samples were then collected and stored at −80 °C until HPLC analysis.

#### 3.7.3. Data Analysis

The PKSolver [24] was used to perform a non-compartmental pharmacokinetics analysis.

### 3.8. In Vitro Metabolic Stability Study in Human Liver Microsomes

#### 3.8.1. Microsomal Incubation

The incubation experiments were performed in duplicates to determine the metabolic stability of capmatinib in human liver microsomes. The microsomal solution contained 7.5 µM capmatinib and human liver microsomes (0.3 mg/mL) in 100 mM of phosphate buffer. We incubated the mixture at 37 °C for 5 min before adding 50 µL of solution A (1.3 mM NADP+, 3.3 mM glucose-6-phosphate, and 3.3 mM magnesium chloride) and solution B (0.4 U/mL glucose-6-phosphate dehydrogenase) to initiate the reaction. Aliquots (100 µL) of the incubation solution were then removed at 0, 3, 6, 10, 20, 30, 45, and 60 min and added to 100 µL of ice-cold methanol containing internal standard naproxen (working solution II) to terminate the reaction. The mixture was then centrifuged at 10,000 rpm for 5 min at 4 °C and 100 µL of the supernatant was transferred into a tube containing 90 µL of ammonium formate buffer (pH 3.5) and mixed before HPLC analysis. A control sample containing no NADPH was also prepared and analyzed.

#### 3.8.2. Data Analysis

The GraphPad Prism (version 8) was used to plot the metabolic curve of the capmatinib and the in vitro pharmacokinetics parameters, the half-life (t_1/2_), and the intrinsic clearance.

## 4. Conclusions

We reported in this research two HPLC methods for studying the pharmacokinetics of capmatinib in vivo and in vitro. To the best of our knowledge, this is the first time HPLC-FLD was used as a sensitive and selective tool to quantify the plasma levels of capmatinib in a rat model and to determine its PK profile following oral administration. The developed method proved to be reliable and fit for its purpose, as demonstrated by the successful validation results according to the EMA guidelines and its application for the determination of capmatinib levels in vivo in a rat animal model. In addition, the HPLC-FLD method demonstrated adequate sensitivity in quantifying 19.5 ng/mL of capmatinib using only 100 μL of plasma volume. The method has the potential to improve the sensitivity further by reducing the dilution volume used in the sample preparation before the sample analysis, thus allowing the quantification of lower concentrations or the use of a smaller sample volume. The sensitivity and low instrument capital cost of the fluorescence detector combined with the simple sample preparation and the short run-time of the chromatographic separation make the HPLC-FLD method ideal for carrying out pharmacokinetic and preclinical studies of capmatinib. We also employed a validated HPLC-PDA as a simple and commonly used technique for studying the metabolic stability of capmatinib in vitro in human liver microsomes. The in vitro metabolic parameters of capmatinib were determined successfully and were comparable with those obtained by LC-MS, thus demonstrating the reliability of the method for conducting capmatinib in vitro experiments. The analytical techniques developed in this work represent simple and sensitive assays that can be used in research and routine analytical labs for carrying out PK studies such as the investigation of potential drug interactions or the obtaining of the pharmacokinetic parameters necessary for in silico modeling purposes.

## Figures and Tables

**Table 1:** Chromatographic parameters of the proposed HPLC-FLD method.

Analyte	Tailing Factor	Retention Time (t_R_) min	Number of Theoretical Plates (N)	Capacity Factor	Selectivity Factor	Resolution
Capmatinib	1.26	1.45	1644	4.61	2.27	7.87
Warfarin	1.20	2.73	1486	10.67		7.99

**Table 2:** Chromatographic parameters of the HPLC-PDA method.

Analyte	Tailing Factor	Retention Time (t_R_) min	Number of Theoretical Plates (N)	Capacity Factor	Selectivity Factor	Resolution
Capmatinib	1.36	3.68	5996	2.82	2.84	5.05
Naproxen	1.03	8.88	11,447	8.02		19.88

**Table 3:** Analytical parameters of the HPLC-FLD method used for quantification of capmatinib in rat plasma.

Linear Range (ng/mL)	Slope Mean ± SD	Intercept Mean ± SD	Correlation Coefficients Mean ± SD
19.5–3920	6.76 ± 4.40	0.0066 ± 0.0028	0.9933 ± 0.0022

**Table 4:** Intra- and inter-day precision and accuracy of the HPLC-FLD method used for the determination of the capmatinib levels in rat plasma.

	Nominal Concentration (ng/mL)	Intra-Day (Single Batch) ^a^			Inter-Day (Three Batches) ^b^		
		Measured Concentration	e_r_ (%)	RSD (%)	Measured Mean Concentration	e_r_ (%)	RSD (%)
LLOQ	19.5	20.45	6.41	6.66	18.77	9.78	11.83
QCL	58.5	59.51	8.06	9.57	55.71	10.96	10.54
QCM	1470	1394.25	5.15	4.15	1469.00	5.55	7.25
QCH	3136.5	2893.19	7.76	4.60	3,082.44	7.10	8.55

^a^*n* = 6, ^b^ *n* = 13.

**Table 5:** Analytical parameters of the developed HPLC-PDA method for capmatinib.

Linear Range (ng/mL)	Slope Mean ± SD	Intercept Mean ± SD	Correlation Coefficients Mean ± SD
206–4124	1.026 ± 0.073	0.03 ± 0.019	0.992 ± 0.0012

^a^*n* = 6.

**Table 6:** Intra- and inter-day precision and accuracy of the method used for determination of capmatinib in HLMs.

	Nominal Concentration (ng/mL)	Intra-Day (Single Batch) ^a^			Inter-Day (Three Batches) ^b^		
		Measured Concentration	e_r_ (%)	RSD (%)	Measured Mean Concentration	e_r_ (%)	RSD (%)
LLOQ	206	210	2.0	3.8	202	4	8.9
QCL	618.6	660	6.6	2.2	635.1	2.7	7.7
QCM	2000	2316	12.4	1.9	2185	6	8.7
QCH	3093	3500	14	0.9	3270	6.4	9.0

^a^*n* = 5, ^b^ *n* = 15.

**Table 7:** Capmatinib pharmacokinetic parameters after the administration of a single oral dose of 10 mg/kg in SD rats.

Parameter	Unit	Value (Mean ± SD)
t_1/2_	h	3.57 ± 1.16
T_max_	h	0.67 ± 0.26
C_max_	ng/mL	1636.55 ± 509.62
${\mathrm{AUC}}_{0}^{t}$	ng/mL.h	8150.59 ± 3754.15
${\mathrm{AUC}}_{0}^{\inf}$	ng/mL.h	8383.64 ± 3639.34
${\mathrm{MRT}}_{0}^{\inf}$	h	4.83 ± 1.4
Vz/F	L	1.83 ± 1.22
Cl/F	L/min/kg	0.03 ± 0.02

## Data Availability

The data presented in this study are available on request from the corresponding author.
